# Pretreatment thrombocytosis predict poor prognosis in patients with endometrial carcinoma: a systematic review and meta-analysis

**DOI:** 10.1186/s12885-018-5264-y

**Published:** 2019-01-15

**Authors:** Dan Nie, E. Yang, Zhengyu Li

**Affiliations:** 1 Department of Obstetrics and Gynecology, Key Laboratory of Birth Defects and Related Diseases of Women and Children, Ministry of Education, West China Second University Hospital, Sichuan University, No. 20 Section 3, Renmin South Road, Chengdu, Sichuan 610041 People’s Republic of China; 2 Department of Obstetrics and Gynecology, The affiliated hospital of Southwest Medical University, Luzhou, 646000 People’s Republic of China

**Keywords:** Thrombocytosis, Prognosis, Endometrial carcinoma

## Abstract

**Background:**

Several previous studies have confirmed that thrombocytosis was related to reduced survival in many solid tumors. However, the prognostic significance of thrombocytosis in endometrial carcinoma (EC) was still controversy. Therefore, we conducted this study to assess the prognostic value of thrombocytosis in EC.

**Methods:**

The database including PubMed, MEDLINE, EMBASE, and Web of Science was searched to explore available literature. Above all, the hazard ratio (HR), odds ratios (OR) with 95% confidence intervals (CIs) was used to investigate the correlation between thrombocytosis and overall survival (OS) and disease-free survival (DFS). Moreover, the association between thrombocytosis and patient clinicopathological characteristics was explored. Publication bias and sensitivity analysis also were conducted in this study.

**Results:**

Overall, 11 studies involving 3439 patients were contained in this study. The results revealed that pretreatment thrombocytosis was significantly related to a decreased OS (pooled HR = 2.99; 95% CI = 2.35–3.8; *P <* 0.001) and DFS (pooled HR = 2.86; 95% CI = 2.27–3.6; *P* <  0.001) in patients with EC. Moreover, thrombocytosis was correlated with adverse clinicopathological parameters.

**Conclusions:**

Pretreatment thrombocytosis is an adverse prognostic marker in patients with EC.

**Electronic supplementary material:**

The online version of this article (10.1186/s12885-018-5264-y) contains supplementary material, which is available to authorized users.

## Background

Endometrial carcinoma (EC) remains one of the most common gynecological cancer in developed countries [1]. In China, EC is the third most common female reproductive system malignancy [2]. According to the previous study, the 5-year overall survival (OS) in EC patients with International Federation of Gynecology and Obstetrics (FIGO) stages I-II are 74–91% [3]. However,10–20% early-stages (I-II) and 50–70% advanced-stage (III-IV) patients will recur after primary treatment [4]. Therefore, it is urgently necessary to explore biomarkers that can be used to tailor distinct treatment protocols, identify high-risk recurrence patients, guide postoperative therapy, and determine follow-up protocols.

Previous studies have revealed that clinicopathological parameters such as histologic type and grade, FIGO stage, myometrial invasion, lymph node (LN) metastasis, lymphovascular space invasion (LVSI), tumor size, and the patients’ age has prognostic effect in patients with EC [5, 6]. However, these factors usually obtained postoperation and demonstrated to be insufficient to predict recurrence and estimate survival [5, 7, 8]. Thus, it is necessary to recognize more effective prognostic predictors to identify high-risk patients preoperation.

Thrombocytosis, often defined as platelet count higher than 400 × 10^9^/L, has been observed correlate with prognosis in various malignancies such as lung, renal, gastric, colorectal and hepatocellular cancer [9–13]. In gynecologic malignancies, pretreatment thrombocytosis was associated with decreased survival in ovarian, vulvar and cervical cancers [14–16]. The prognostic role of thrombocytosis in EC also has been reported in several studies, but the conclusions were controversy [17–29]. Therefore, we conducted this meta-analysis to elucidate the prognostic value of pretreatment thrombocytosis in EC.

## Methods

### Search strategy

We identified relevant studies by searching database including PubMed, MEDLINE, EMBASE, and Web of Science. The search was updated in August 2018. The search strategy was as follows: (((((((“Endometrial Neoplasms”[Mesh]) OR endometrial cancer) OR endometrial carcinoma) OR endometrium cancer) OR endometrium carcinoma)) AND (((((((“Thrombocytosis”[Mesh]) OR thrombocytosis) OR thrombocythemia) OR platelet count) OR blood platelets) OR platelets) OR platelet)) AND (((((“Prognosis”[Mesh]) OR prognosis) OR prognostic) OR survival) OR mortality). This study was performed following the Preferred Reporting Items for Systematic Reviews and Meta-Analyses (PRISMA) guidelines (Additional file 1) [30].

### Selection criteria

The including criteria were as follows: (1) EC was diagnosed by histopathological examination; (2) platelet count was measured preoperation; (3) hazard ratios(HRs) and their 95% confidence intervals (CIs) for platelet count can be obtained; (4) the cut-off value of thrombocytosis was provided.

### Exclusion criteria

The exclusion criteria were as follows: (1) letters, meeting abstracts, reviews; (2) articles not written in English; (3) studies with duplicate data; (4) incomplete data for evaluating the HR and its 95%CI. The candidate articles were assessed by two reviewers independently. Any disputes were resolved through their discussion.

### Data extraction and quality assessment

In the light of the guidelines for meta-analysis of observational studies [31], two reviewers independently extracted data from the selected literature. The obtained data were as follows: the first author name, study publication time, country, sample size, FIGO stage, grade, LVSI, histological type, LN metastasis, recurrence, the cut-off value of thrombocytosis, venous thromboembolism (VTE), follow-up time, and primary outcome. The Newcastle-Ottawa Scale (NOS) scoring system was used to assess the quality of selected articles [32]. High-quality studies were defined as NOS score more than six (Additional file 2).

### Statistical analysis

The HRs and 95% CIs were used to evaluate the prognostic significance of thrombocytosis on EC. Additionally, the correlation between thrombocytosis and clinicopathological characteristics were analyzed. Heterogeneity analysis was assessed using Chi-square test based on the *Q* value and *I*^*2*^ statistic value. Using a random-effects model, or using a fixed-effects model was determined by the level of heterogeneity (e.g., *P*-value < 0.1 and/or *I*^*2*^ > 50%, the random-effects model was used). Besides, we conducted a sensitivity analysis to validate the stability of the pooled outcomes. Begg’s test and Egger’s test was used to assessing the publication bias. The data in our study were analyzed using STATA 14.0 software (Stata Corporation, College Station, TX, USA).

## Results

### Characteristics of eligible studies

**Fig. 1:**
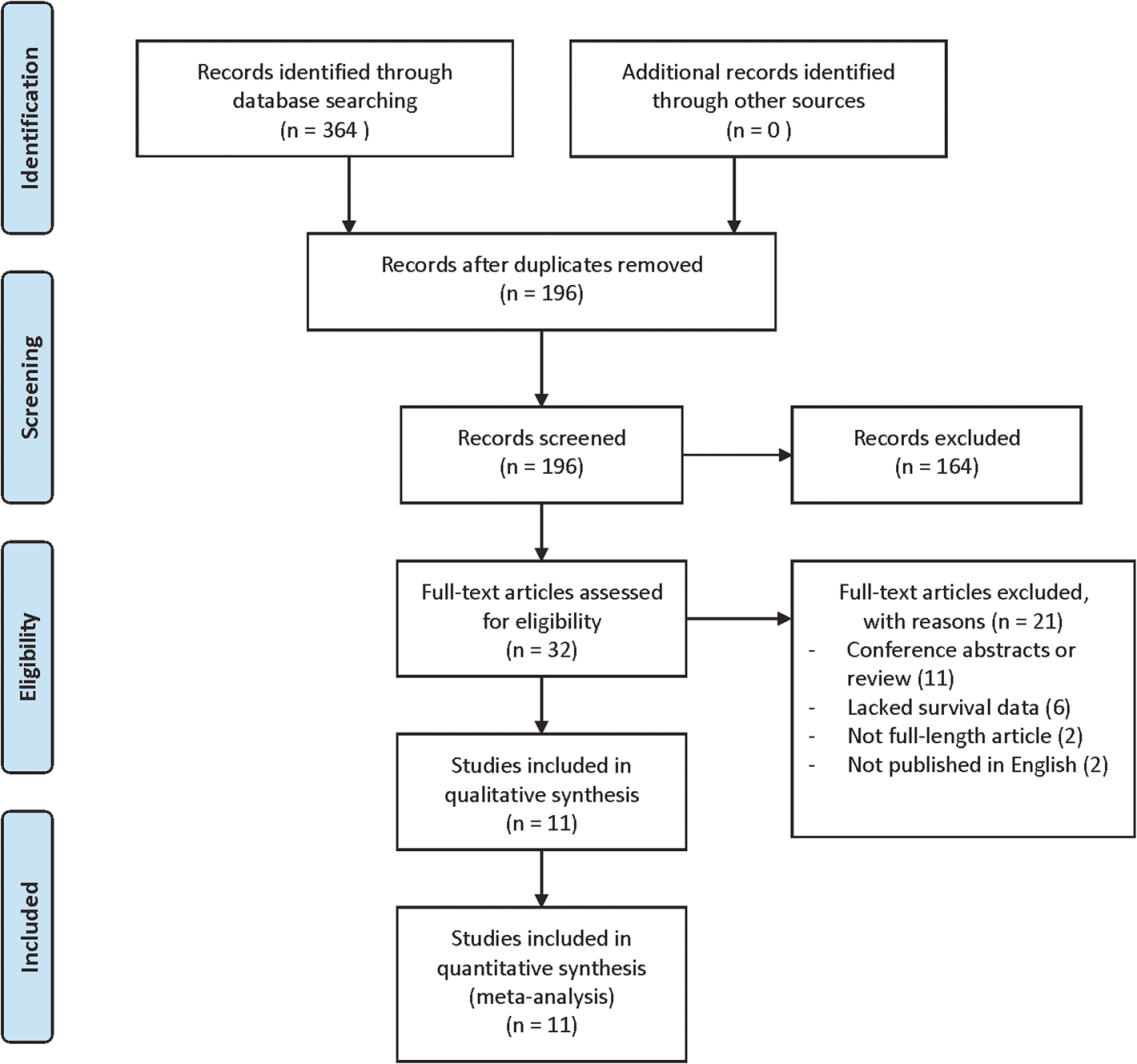
The flow diagram of the study selection strategy

The flow diagram illustrated the search procedure (Fig. 1). After the preliminary search, we identified a total of 364 articles. First, we removed 168 duplicate articles, and another 164 irrelevant items also were excluded. The remaining 32 full-text articles were left for further review. Among them, six studies were excluded due to lack of survival data. Next, we excluded two non-English articles, 11 conference abstracts or reviews, and two not full-length articles. Finally, 11 eligible studies were involved in this meta-analysis. All of the qualified studies were observational retrospective studies. The main characteristics of the eligible studies were summarized in Table 1.

**Table 1:** Main characteristics of the eligible studies included in the meta-analysis

Author	Year	Country	Patients number	Age (years)	FIGO Stage	Tumor Grade	Tumor type	cut-off value (× 10^9^/L)	Incidence of thrombocytosis (%)	Follow-up time (month)	Primary Outcome	NOS
Abu-Zaid [24]	2017	Saudi Arabia	162	59	I–IV	1–3	endometrioid	400	8.6	NR	OS, DFS	6
Andersen [27]	2017	Denmark	218	18–80	I–IV	1–3	mixed	400	11.5	NR	OS, DSS	6
Njølstad [28]	2013	Norway	512	28–93	I–IV	1–3	mixed	390	12.3	55(0–97)	DFS	6
Kizer [22]	2015	USA	318	62	I–IV	1–3	mixed	400	16.7	NR	DFS, DSS	6
Nakamura [23]	2016	Japan	108	60	I–IV	1–3	mixed	350	11.02	NR	OS, PFS	6
Takahashi [26]	2017	Japan	508	58	I–IV	1–3	mixed	400	7	NR	OS	6
Heng [20]	2014	Thailand	238	28–88	I–IV	1–3	mixed	400	18.1	59.6(1–98)	OS, DFS	7
Matsuo [19]	2013	USA	516	52	I–IV	1–3	mixed	400	15.1	43.7	OS, DFS	8
Gorelick [18]	2009	USA	77	65	I–IV	1–3	mixed	400	18.2	NR	OS	6
Lerner [17]	2007	USA	68	NR	III-IV	NR	serous	400	12	NR	OS	6
Moeini [25]	2017	USA	714	53.1	I–IV	1–3	mixed	400	24.8	28.8	OS, DFS	8

*Abbreviations*: *NOS* Newcastle Ottawa Scale, *NR* not reported, *FIGO* International Federation of Gynecology and Obstetrics, *OS* overall survival, *PFS* progression-free survival, *DFS* disease-free survival, *DSS* disease-specific survival

### The prognostic impact of thrombocytosis on overall survival

**Fig. 2:**
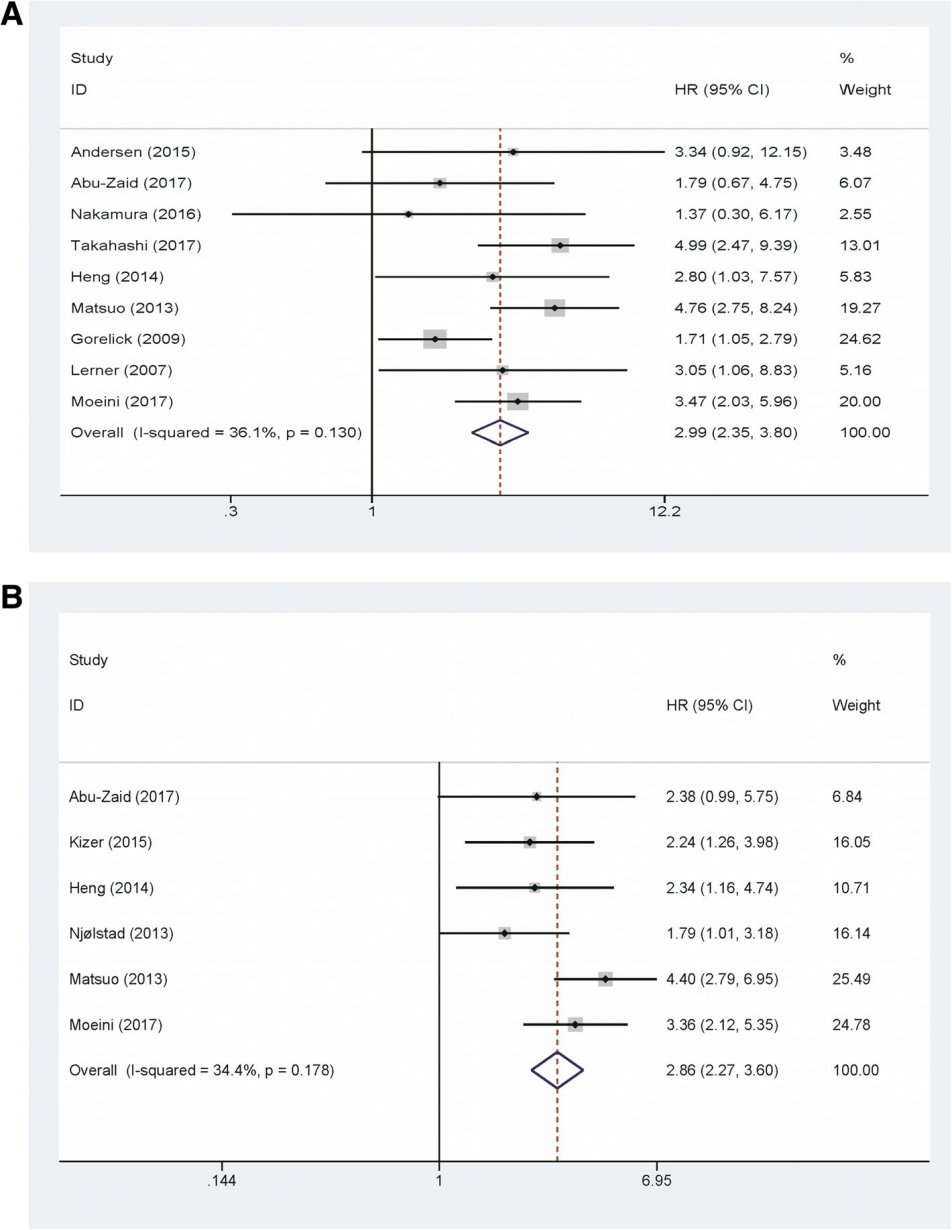
**a** Forest plot to assess the association between thrombocytosis and overall survival. **b** Forest plot to assess the association between thrombocytosis and disease-free survival

A total of nine studies including 2609 EC patients provided the overall survival data for analysis. The results revealed that elevated platelet count correlated with poor OS in EC patients (pooled HR = 2.99; 95% CI = 2.35–3.8; *P <* 0.001) (Fig. 2a). Subgroup analysis was conducted to further investigate the prognostic role of thrombocytosis on OS in patients with EC. We observed significant results in subgroup analysis based on the study region (Asian vs. Non-Asian), sample size (< 200 vs. ≥ 200), FIGO stage (I-IV vs. III-IV), and analysis method (Multivariate vs. Univariate). In subgroup analysis according to the cut-off value of platelet count, a significant result was observed in the platelet count = 400 × 10^9^/L group (HR = 3.05, 95% CI = 2.39–3.89, fixed effects). However, platelet count = 350 × 10^9^/L group did not predict poor prognosis for EC (HR = 1.37,95% CI = 0.3–6.17, fixed effects) (Table 2).

**Table 2:** Summary of subgroup analysis

Categories	Number of studies	Number of patients	Model	HR (95% CI)	*I*^*2*^ (%)	*P*_*h*_	*Z*	*P*
Study region								
Asian	4	1016	Fixed	3.12 (0.54–5.20)	31	0.23	4.85	< 0.001
Non-Asian	5	1593	Random	3.03 (1.96–4.69)	51	0.09	4.98	< 0.001
Sample size								
< 200	4	415	Fixed	1.84 (1.25–2.71)	0	0.78	3.07	0.002
≥ 200	5	2194	Fixed	4.04 (2.98–5.50)	0	0.8	8.92	< 0.001
FIGO stage								
I–IV	8	2541	Fixed	2.98 (2.33–3.82)	44	0.08	8.66	< 0.001
III–IV	1	68	Fixed	3.05 (1.06–8.80)	NA	NA	2.06	0.04
Cut-off value (×10^9^/L)								
350	1	108	Fixed	1.37 (0.30–6.17)	NA	NA	0.41	0.68
400	8	2501	Fixed	3.05 (2.39–3.89)	39	0.12	8.95	< 0.001
Analysis method								
Multivariate	5	1053	Fixed	2.47 (1.78–4.42)	43	0.13	5.44	< 0.001
Univariate	4	1556	Fixed	3.76 (2.63–5.37)	0	0.46	7.25	< 0.001

Random-effects model was used when *P*-value for heterogeneity test < 0.1; otherwise, fixed-effects model was used

*Abbreviations*: *FIGO* International Federation of Gynecology and Obstetrics, *HR* hazard ratio, *CI* confidence interval, *P*_*h*_, *P*-value for heterogeneity based on *Q* test, *P P*-value for statistical significance based on *Z* test, *NA* Not applicable

### The prognostic impact of thrombocytosis on disease-free survival

Six studies contain 2460 patients were included to evaluate the correlation between thrombocytosis and DFS in EC patients. The result showed that thrombocytosis predicted a worse DFS in the fixed effects model (pooled HR = 2.86; 95% CI = 2.27–3.6; *P* <  0.001) (Fig. 2b).

### Correlation between thrombocytosis and clinicopathological characteristics in patients with EC

We further analyzed the correlation between thrombocytosis and clinicopathologic characteristics (Table 3)**.** Thrombocytosis was positively related to FIGO stage (odds ratio (OR) = 3.24; 95% CI =1.78–5.88; *P* <  0.001), tumor grade (OR = 1.89; 95% CI =1.3–2.76; *P* = 0.001), LN metastasis (OR = 3.13; 95% CI =1.71–5.75; *P* <  0.001), LVSI (OR = 1.98, 95% CI =1.34–2.94; *P* = 0.001), cancer recurrence (OR = 8.57; 95% CI =3.71–19.83; *P <* 0.001), and VTE (OR = 6.45; 95% CI =4.06–10.24; *P* <  0.001). However, thrombocytosis was not associated with histologic type of EC (OR = 1.45; 95% CI = 0.76–2.77; *P* = 0.265).

**Table 3:** Analysis of the association between thrombocytosis and clinicopathological parameters of endometrial carcinoma

Clinicopathological characteristics	Number of studies	Model	OR (95% CI)	*I*^*2*^ (%)	*P*_*h*_	*Z*	*P*
FIGO stage (III-IV vs. I-II)	5	Random	3.24 (1.78–5.88)	58.1	0.049	3.86	< 0.001
Grade (2–3 vs.1)	6	Fixed	1.89 (1.30–2.76)	19.4	0.287	3.32	0.001
LVSI (yes vs. no)	5	Fixed	1.98 (1.34–2.94)	0	0.603	3.4	0.001
Histological type (non-endometrioid vs. endometrioid)	5	Random	1.45 (0.76–2.77)	60.6	0.038	1.11	0.265
LN metastasis (yes vs. no)	2	Fixed	3.13 (1.71–5.75)	0	0.416	3.69	< 0.001
Recurrence (yes vs. no)	2	Fixed	8.57 (3.71–19.83)	0	0.458	5.02	< 0.001
VTE (yes vs. no)	2	Fixed	6.45 (4.06–10.24)	0	0.807	7.91	< 0.001

Random-effects model was used when *P*-value for heterogeneity test < 0.1; otherwise, fixed-effects model was used

*Abbreviations*: *FIGO* International Federation of Gynecology and Obstetrics, *LVSI* lymphovascular space invasion, *LN* lymph node, *VTE* venous thromboembolism, *OR* odds ratio, *CI* confidence interval, *P*_*h*_, *P*-value for heterogeneity based on *Q* test, *P P*-value for statistical significance based on *Z* test

### Publication bias and sensitivity analysis

**Fig. 3:**
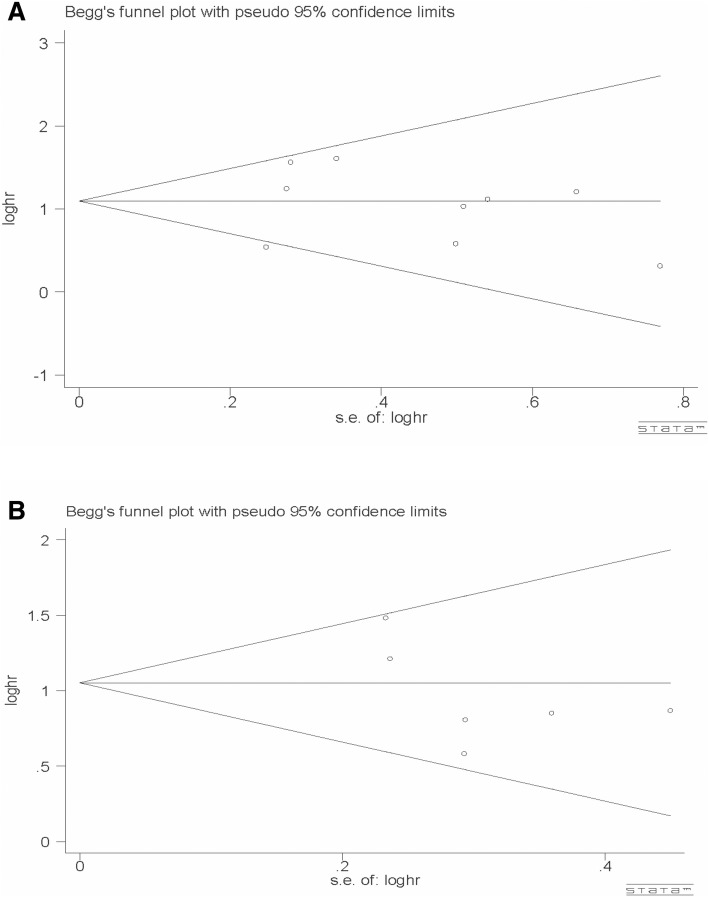
Funnel plot of publication bias for overall survival (**a**) and disease-free survival (**b**)

**Fig. 4:**
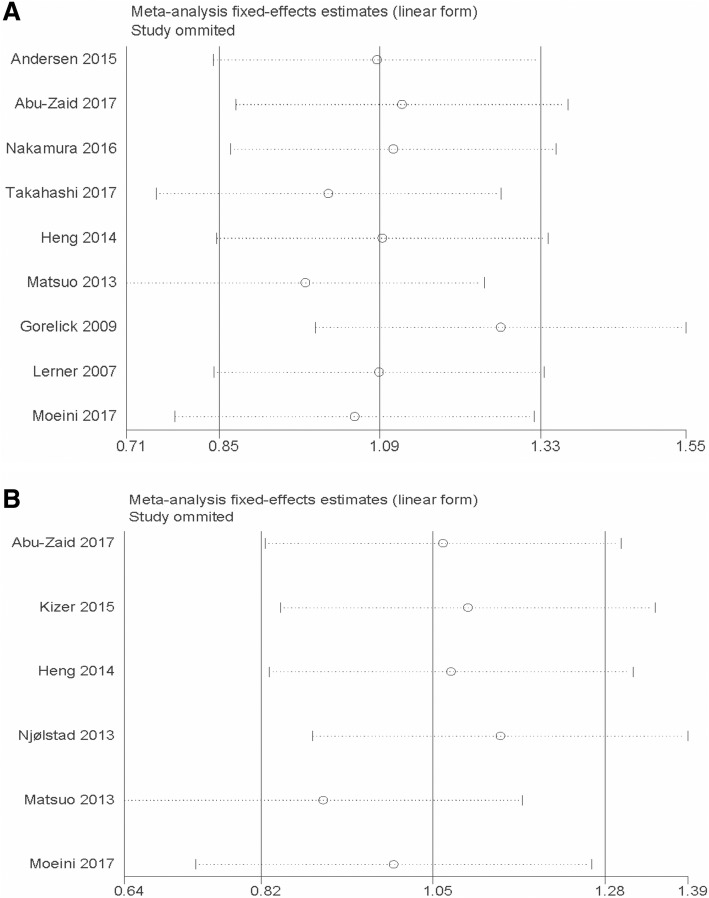
Sensitivity analysis of the association between thrombocytosis and overall survival (**a**) and disease-free survival (**b**)

There was no apparent publication bias for OS (*P* = 0.917 for Begg’s test and *P* = 0.740 for Egger’s test) and DFS (*P* = 0.707 for Begg’s test and *P* = 0.192 for Egger’s test) (Fig. 3) in our study. Additionally, a sensitivity analysis was carried out by sequentially omitting eligible studies. The results confirmed the stability and reliability of the outcomes (Fig. 4).

## Discussion

The critical role of platelets in inflammatory and immune responses has been confirmed [33]. Several studies have indicated that elevated platelets play crucial roles in promoting cancer growth and metastasis [14]. The interactions between platelets and cancer cells activate TGFβ/Smad and NF-κB pathways, subsequently inducing the occurrence of epithelial-mesenchymal transition and promoting cancer metastasis [34, 35]. Platelets promote cancer metastasis also depending on the activating of YAP1 signaling through the RhoA / MYPT-PP1 pathway [36]. Additionally, activated platelets lead to the release of tumor growth factors and chemokines, such as platelet-derived growth factor (PDGF), vascular endothelial growth factor (VEGF) and CXCL5 and CXCL7, which stimulate cancer growth and metastasis [37, 38]. Furthermore, platelets protect cancer cells from the immune clearance by natural killer cells, which also accelerates cancer metastasis [38]. Thus, there exists a complex cross-talk between platelets and cancers.

The elevated platelet counts more than 400 × 10^9^/L often defined as thrombocytosis. Thrombocytosis has been proved to correlate with adverse prognosis in many malignancies. The incidence rate of pretreatment thrombocytosis was range from 7 to 24.8% in the included studies. Our results demonstrated that pretreatment thrombocytosis predicts a worse OS and DFS in patients with EC. In subgroup analysis, we showed that the elevated platelet count also reveals a decreased OS in patients with EC except in subgroup analysis by platelet count cut-off value. Using 350 × 10^9^/L as platelet count cut-off value did not predict a poor prognosis.

Moreover, we investigated the association between thrombocytosis and clinicopathological characteristics in EC patients. According to our findings, pretreatment thrombocytosis was significantly associated with advanced FIGO stage, tumor grade, LVSI, LN metastasis, recurrence, and VTE. However, pretreatment thrombocytosis was not related to the histologic types.

Our meta-analysis also has some flaws. First, EC patients mostly accompanied by menorrhagia or abnormal uterine bleeding, which always leads to anemia [5]. Patients with thrombocytosis commonly coexist with iron deficiency anemia [39]. The included studies did not classify whether anemia-associated thrombocytosis or paraneoplastic thrombocytosis was related to prognosis. That may lead to potential confounding. Second, all of the included studies were retrospective studies, which may cause selection biases. Third, the cut-off values for definition of thrombocytosis differed in the included studies. Most of the studies used 400 × 10^9^/L as cut-off value of platelet count to diagnose thrombocytosis. However, one of the studies used 350 × 10^9^/L as the platelet count cut-off value [24]. The distinct cut-off value may lead to apparent heterogeneity between studies. Thus, establishing a consistent platelet count cutoff value to diagnose thrombocytosis is necessary. Last but not least, several factors such as patients’ age, tumor size, adjuvant therapy, which will affect platelet count did not include in our analysis. Therefore, more studies are needed to verify our findings.

## Conclusions

In conclusion, this systematic review demonstrated that pretreatment thrombocytosis is correlated with poor survival outcome and adverse clinicopathological parameters in EC, and thrombocytosis is a potential prognosis predictor for EC.

## Additional files

Additional file 1: The PRISMA 2009 checklist. (DOC 66 kb)

Additional file 2: The Newcastle-Ottawa quality assessment scale. (DOCX 16 kb)

## Abbreviations

CI: confidence interval
DFS: disease-free survival
EC: endometrial carcinoma
FIGO: International Federation of Gynecology and Obstetrics
HR: hazard ratio
LN: lymph node
LVSI: lymphovascular space invasion
OR: odds ratio
OS: overall survival
VTE: venous thromboembolism

## References

[1] Siegel RL, Miller KD, Jemal A (2016). Cancer statistics, 2016. CA Cancer J Clin.

[2] Chen W, Zheng R, Baade PD (2016). Cancer statistics in China, 2015. CA Cancer J Clin.

[3] Murali R, Soslow RA, Weigelt B (2014). Classification of endometrial carcinoma: more than two types. Lancet Oncol.

[4] Lewin SN, Herzog TJ, Barrena MNI (2010). Comparative performance of the 2009 international federation of gynecology and obstetrics' staging system for uterine corpus cancer. Obstet Gynecol.

[5] Morice P, Leary A, Creutzberg C, Abu-Rustum N, Darai E (2016). Endometrial cancer. Lancet.

[6] Nie D, Zhang L, Guo Q, Mao X (2018). High mobility group protein A2 overexpression indicates poor prognosis for cancer patients: a meta-analysis. Oncotarget.

[7] Salvesen HB, Haldorsen IS, Trovik J (2012). Markers for individualised therapy in endometrial carcinoma. Lancet Oncol.

[8] Tejerizo-García A, Jiménez-López JS, Muñoz-González JL (2013). Overall survival and disease-free survival in endometrial cancer: prognostic factors in 276 patients. Onco Targets Ther.

[9] Zhang X, Ran Y (2015). Prognostic role of elevated platelet count in patients with lung cancer: a systematic review and meta-analysis. Int J Clin Exp Med.

[10] Bensalah K, Leray E, Fergelot P (2006). Prognostic value of thrombocytosis in renal cell carcinoma. J Urol.

[11] Wang YH, Kang JK, Zhi YF (2018). The pretreatment thrombocytosis as one of prognostic factors for gastric cancer: a systematic review and meta-analysis. Int J Surg.

[12] Josa V, Krzystanek M, Eklund AC, Salamon F, Zarand A, Szallasi Z, Baranyai Z (2015). Relationship of postoperative thrombocytosis and survival of patients with colorectal cancer. Int J Surg.

[13] Pang Q, Qu K, Zhang JY (2015). The prognostic value of platelet count in patients with hepatocellular carcinoma: a systematic review and meta-analysis. Medicine (Baltimore).

[14] Stone RL, Nick AM, McNeish IA (2012). Paraneoplastic thrombocytosis in ovarian cancer. N Engl J Med.

[15] Lavie O, Comerci G, Daras V, Bolger BS, Lopes A, Monaghan JM (1999). Thrombocytosis in women with vulvar carcinoma. Gynecol Oncol.

[16] Zhao K, Deng H, Qin Y, Liao W, Liang W (2015). Prognostic significance of pretreatment plasma fibrinogen and platelet levels in patients with early-stage cervical cancer. Gynecol Obstet Investig.

[17] Gucer F, Moser F, Tamussino K, Reich O, Haas J, Arikan G, Petru E, Winter R (1998). Thrombocytosis as a prognostic factor in endometrial carcinoma. Gynecol Oncol.

[18] Lerner DL, Walsh CS, Cass I, Karlan BY, Li AJ (2007). The prognostic significance of thrombocytosis in uterine papillary serous carcinomas. Gynecol Oncol.

[19] Gorelick C, Andikyan V, Mack M, Lee YC, Abulafia O (2009). Prognostic significance of preoperative thrombocytosis in patients with endometrial carcinoma in an inner-city population. Int J Gynecol Cancer.

[20] Matsuo K, Wu E, Yessaian A, Lin Y, Pham H, Muderspach L, Liebman H, Morrow C, Roman L (2013). Predictive model of venous thromboembolism in endometrial cancer. Gynecologic OncologyGynecol Oncol.

[21] Heng S, Benjapibal M (2014). Preoperative thrombocytosis and poor prognostic factors in endometrial cancer. Asian Pac J Cancer Prev.

[22] Kaloglu S, Guraslan H, Tekirdag AI, Dagdeviren H, Kaya C (2014). Relation of preoperative thrombocytosis between tumor stage and grade in patients with endometrial Cancer. Eurasian J Med.

[23] Kizer NT, Hatem H, Nugent EK, Zhou G, Moore K, Heller P, Mutch DG, Thaker PH (2015). Chemotherapy response rates among patients with endometrial Cancer who have elevated serum platelets. Int J Gynecol Cancer.

[24] Nakamura K, Nakayama K, Ishikawa M (2016). High pretreatment plasma D-dimer levels are related to shorter overall survival in endometrial carcinoma. Eur J Obstet Gynecol Reprod Biol.

[25] Abu-Zaid A, Alsabban M, Abuzaid M, AlOmar O, Salem H, Al-Badawi IA (2017). Preoperative thrombocytosis as a prognostic factor in endometrioid-type endometrial carcinoma. Ann Saudi Med.

[26] Moeini A, Machida H, Takiuchi T, Blake EA, Hom MS, Miki T, Matsuo O, Matsuo K (2017). Association of Nonalcoholic Fatty Liver Disease and Venous Thromboembolism in women with endometrial Cancer. Clin Appl Thromb Hemost.

[27] Takahashi R, Mabuchi S, Kuroda H (2017). The significance of pretreatment thrombocytosis and its association with neutrophilia in patients with surgically treated endometrial Cancer. Int J Gynecol Cancer.

[28] Andersen CL, Eskelund CW, Siersma VD, Felding P, Lind B, Palmblad J, Bjerrum OW, Friis S, Hasselbalch HC, de Fine Olivarius N (2015). Is thrombocytosis a valid indicator of advanced stage and high mortality of gynecological cancer. Gynecol Oncol.

[29] Njølstad TS, Engerud H, Werner HM, Salvesen HB, Trovik J (2013). Preoperative anemia, leukocytosis and thrombocytosis identify aggressive endometrial carcinomas. Gynecol Oncol.

[30] Moher D, Liberati A, Tetzlaff J, Altman DG (2009). Preferred reporting items for systematic reviews and meta-analyses: the PRISMA statement. BMJ.

[31] Stroup DF, Berlin JA, Morton SC (2000). Meta-analysis of observational studies in epidemiology: a proposal for reporting. Meta-analysis of observational studies in epidemiology (MOOSE) group. JAMA.

[32] Stang A (2010). Critical evaluation of the Newcastle-Ottawa scale for the assessment of the quality of nonrandomized studies in meta-analyses. Eur J Epidemiol.

[33] Semple JW, Italiano JE, Freedman J (2011). Platelets and the immune continuum. Nat Rev Immunol.

[34] Labelle M, Begum S, Hynes RO (2011). Direct signaling between platelets and cancer cells induces an epithelial-mesenchymal-like transition and promotes metastasis. Cancer Cell.

[35] Cho MS, Bottsford-Miller J, Vasquez HG (2012). Platelets increase the proliferation of ovarian cancer cells. Blood.

[36] Haemmerle M, Taylor ML, Gutschner T (2017). Platelets reduce anoikis and promote metastasis by activating YAP1 signaling. Nat Commun.

[37] Li N (2016). Platelets in cancer metastasis: to help the "villain" to do evil. Int J Cancer.

[38] Tesfamariam B (2016). Involvement of platelets in tumor cell metastasis. Pharmacol Ther.

[39] Akan H, Güven N, Aydogdu I, Arat M, Beksaç M, Dalva K (2000). Thrombopoietic cytokines in patients with iron deficiency anemia with or without thrombocytosis. Acta Haematol.

