# Supplementary material for: Pretreatment thrombocytosis predict poor prognosis in patients with endometrial carcinoma: a systematic review and meta-analysis
Source: BMC Cancer. 2019 Jan 15;19:73. doi: 10.1186/s12885-018-5264-y (PMC6332560; doi:10.1186/s12885-018-5264-y)
Supplement: Supplementary file 2 — The Newcastle-Ottawa quality assessment scale. (DOCX 16 kb) [file 12885_2018_5264_MOESM2_ESM.docx]

**NEWCASTLE - OTTAWA QUALITY ASSESSMENT SCALE**

| **Study** | **Year** | **Selection** | | | |  | **Comparability** |  | **Outcome** | | | **Score** |
| --- | --- | --- | --- | --- | --- | --- | --- | --- | --- | --- | --- | --- |
|  |  | **Representativeness of the exposed cohort** | **Selection of the non-exposed cohort** | **Ascertainment of exposure** | **Demonstration that outcome of interest was not present at start of study** |  | **Comparability of cohorts on the basis of the design or analysis** |  | **Assessment of outcome** | **Was follow-up long enough for outcomes to occur** | **Adequacy of follow up of cohorts** |  |
| Abu-Zaid | 2017 | ☆ | ☆ | ☆ | ☆ |  |  |  | ☆ | ☆ |  | 6 |
| Andersen | 2017 | ☆ | ☆ | ☆ | ☆ |  |  |  | ☆ | ☆ |  | 6 |
| Njølstad | 2013 | ☆ | ☆ | ☆ | ☆ |  |  |  | ☆ | ☆ |  | 6 |
| Kizer | 2015 | ☆ | ☆ | ☆ | ☆ |  |  |  | ☆ | ☆ |  | 6 |
| Nakamura | 2016 | ☆ | ☆ | ☆ | ☆ |  |  |  | ☆ | ☆ |  | 6 |
| Takahashi | 2017 | ☆ | ☆ | ☆ | ☆ |  |  |  | ☆ |  | ☆ | 6 |
| Heng | 2014 | ☆ | ☆ | ☆ | ☆ |  | ☆ |  | ☆ |  | ☆ | 7 |
| Matsuo | 2013 | ☆ | ☆ | ☆ | ☆ |  | ☆ |  | ☆ | ☆ | ☆ | 8 |
| Gorelick | 2009 | ☆ | ☆ | ☆ | ☆ |  | ☆ |  | ☆ | ☆ |  | 6 |
| Lerner | 2007 | ☆ | ☆ | ☆ | ☆ |  |  |  | ☆ | ☆ |  | 6 |
| Moeini | 2017 | ☆ | ☆ | ☆ | ☆ |  | ☆ |  | ☆ | ☆ | ☆ | 8 |
